# MoS_2_/Au Heterojunction Catalyst for SERS Monitoring of a Fenton-like Reaction

**DOI:** 10.3390/ma16031169

**Published:** 2023-01-30

**Authors:** Qian Wei, Beibei Lu, Qing Yang, Can Shi, Yulan Wei, Minmin Xu, Chenjie Zhang, Yaxian Yuan

**Affiliations:** 1 College of Chemistry, Chemical Engineering and Materials Science, Soochow University, Suzhou 215123, China; 2 Suzhou Key Laboratory of Novel Semiconductor-Optoelectronics Materials and Devices, Soochow University, Suzhou 215123, China

**Keywords:** MoS_2_/Au heterojunctions, Fenton-like reaction, methylene blue, surface enhanced Raman spectroscopy

## Abstract

Fenton technology is one of advanced oxidation process (AOP) methods to treat wastewater through chemical oxidation. Due to the limitations of classical iron-based catalysts, it is still challenging to find suitable catalysts for Fenton-like reactions. Here, MoS_2_/Au heterojunctions were successfully synthesized by reduction of chloroauric acid in the solution of layered MoS_2_ prepared by hydrothermal method. As a model molecule, methylene blue (MB) was used as the species to be degraded to evaluate the performance of the catalyst. It was determined by UV–visible spectra that the optimal catalyst can be obtained when MoS_2_ (mg): HAuCl_4_ (wt. % mL) is 2:2. The Fenton-like reaction process was monitored by introducing highly sensitive surface enhanced Raman spectroscopy (SERS). The results show that MB can be degraded by 83% in the first 10 min of the reaction, indicating that MoS_2_/Au has good catalytic performance. In addition, as a fingerprint spectrum, SERS was used to preliminarily analyze the molecular structure changes during the degradation process. The result showed that C-N-C bond was easier to break than the C-S-C bond. NH_2_ group and the fused ring were destroyed at the comparable speed at the first 30 min. In terms of application applicability, it was showed that MB degradation had exceeded 95% at all the three pH values of 1.4, 5.0, and 11.1 after the reaction was carried out for 20 min. The test and analysis of the light environment showed that the catalytic efficiency was significantly improved in the natural light of the laboratory compared to dark conditions. The possible mechanism based on ·OH and ·O_2_^−^ from ESR data was proposed. In addition, it was demonstrated to be a first-order reaction from the perspective of kinetics. This study made a positive contribution to broaden of the applicable conditions and scope of Fenton-like reaction catalysts. It is expected to be used as a non-iron catalyst in practical industrial applications. From the perspective of detection method, we expect to develop SERS as a powerful tool for the in situ monitoring of Fenton-like reactions, and to further deepen our understanding of the mechanism.

## 1. Introduction

Industrial polluted wastewater needs to be urgently treated with the rapid development of industry [1,2]. Pollutants can be concentered by physical methods such as adsorption, ion exchange, reverse osmosis, and ultrafiltration [3], and can also be degraded by chemical methods. Advanced oxidation process (AOP) is a technology which produces highly oxidizing active intermediates to degrade target pollutants [4]. It can transform stubborn pollutants to smaller degradable intermediates, and finally tear them into water and carbon dioxide in the most ideal circumstances [5].

The Fenton reaction, which belongs to one of the AOP technologies, depends on the electron transfer between hydrogen peroxide (H_2_O_2_) and Fe^2+^. The H_2_O_2_ reacts with Fe^2+^ to form Fe^3+^ and a hydroxyl radical (**·**OH) [4]. The **·**OH has strong oxidizability and is mainly responsible for the degradation of organic pollutants. However, a large amount of iron sludge can be produced, which limits the application of the catalyst. Therefore, it is particularly important to find a candidate for Fenton-like reactions. One is to composite other materials with ferrous materials, for example, Fe-doped Bi_4_O_5_I_2_ microflowers [6], iron-modified zeolitic tuff [7], and Fe-Cu alloy [8]. The other is completely free of iron, such as, CuO [9], metal sulfides [10], and micelle-clay [11]. Specially, Liu’s group prepared single cobalt atom anchored on porous N-doped graphene nanocomposite. The catalyst contained double reaction active sites, which can effectively catalyze the oxidation of refractory organic compounds through the activation of peroxymonosulfate (PMS) [12]. Molybdenum disulfide (MoS_2_) is widely used in optoelectronic devices [13], light-emitting devices [14], and catalysis [15] due to its excellent physical and chemical properties. It was reported that MoS_2_ was used as a cocatalyst in a Fenton/Fenton-like reaction, such as Cu_2_O–MoS_2_ [16], MoS_2_/FeS [17], BiOBr/MoS_2_ [18], Cu-CN/MoS_2_ [19], and NH_2_-MIL-101(Fe)/MoS_2_ [20]. However, the application of MoS_2_ as a main catalyst in Fenton-like reaction is rarely reported. Surface enhanced Raman spectroscopy (SERS) is a kind of vibration spectroscopy with an ultrahigh sensitivity, which can provide fingerprint information for the probe molecules. Therefore, SERS can be used to detect trace substances, study the chemical reaction process, and even find the intermediate products produced in the chemical reaction [21,22,23,24]. Although SERS has been widely used in many research fields, there were rare reports on Fenton-like reaction studies.

In this paper, MoS_2_/Au heterojunction catalysts were prepared by wet chemical reduction of Au nanoparticles on the layered MoS_2_ surface. The adhesion of Au nanoparticles on the surface of MoS_2_ increases the interface defects, thus improving the catalytic performance. At the same time, the addition of Au also makes the catalyst have Raman signal enhancement performance. This dual-functional material has laid a solid foundation for the catalysis and detection of Fenton-like reactions. SERS technology was successfully used in reaction monitoring, and the catalytic efficiency under different conditions (light environment, pH value) was obtained. In addition, it was used in the study of kinetic reaction order. Our research is expected to make contributions to the development of this field in terms of catalysts and detection methods.

## 2. Materials and Methods

### 2.1. Chemicals

Chloroauric acid tetrahydrate (HAuCl_4_·4H_2_O, AR), ascorbic acid (AA, AR), dimethyl sulfoxide (DMSO, AR), and ammonium molybdate tetrahydrate (AMTH, AR) were purchased from Sinopharm Chemical Co., Ltd. (Shanghai, China). 5,5-dimethyl-1-pyrroline n-oxide (DMPO, 97%) was obtained from Shanghai Aladdin Biochemical Technology Co., Ltd. (Shanghai, China). Methylene blue (MB, BS) was derived from Shanghai Yuanye Bio-Technology Co., Ltd. (Shanghai, China). Thiourea (99%) was obtained from Alfa Aesar (Haverhill, MA, USA). Hydrogen peroxide (H_2_O_2_) (30%, aqueous solution, AR) was obtained from Shanghai Lingfeng Chemical Reagent Co., Ltd. (Shanghai, China). All the water used was Milli-Q water (≥18.2 MΩ·cm).

### 2.2. Preparation of MoS_2_ and MoS_2_/Au Nanomaterials

**Scheme 1:**
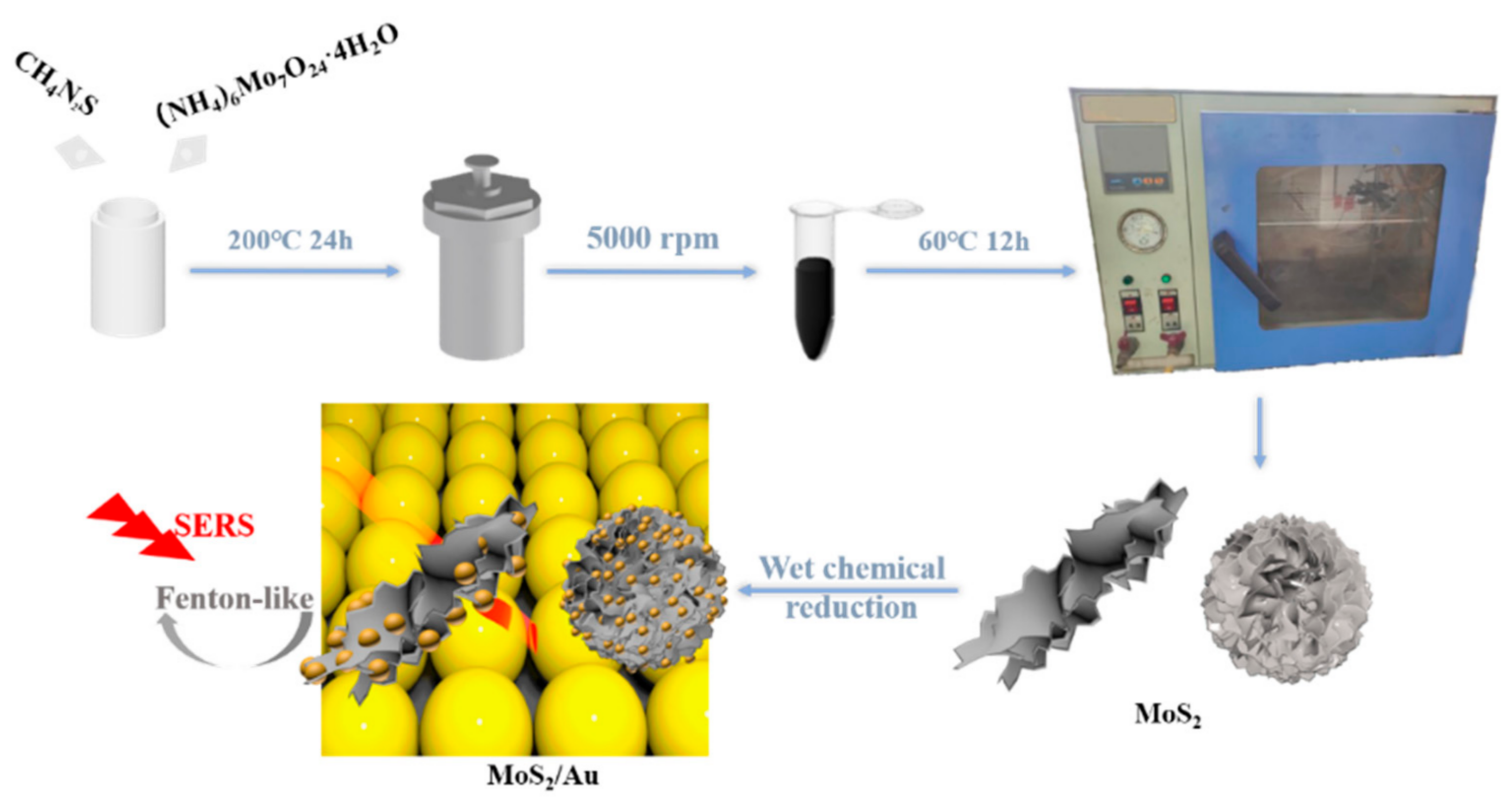
Schematic illustration of layered MoS_2_ and MoS_2_/Au heterojunctions preparation processes for Fenton-like reaction monitored by SERS.

**Figure A1:**
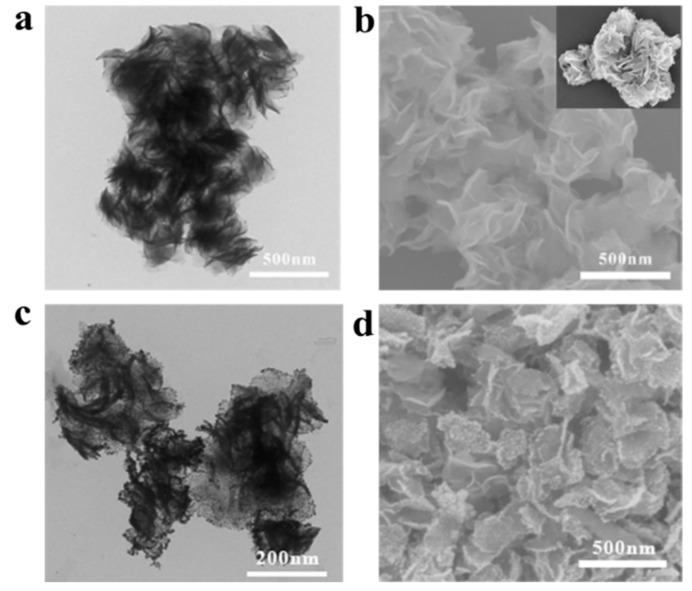
TEM and SEM images of MoS_2_ sheets (**a**,**b**) and MoS_2_/Au heterojunctions (**c**,**d**). The inset presents a SEM image of MoS_2_ microflower.

As shown in Scheme 1, 1.06 mg of AMTH and 1.81 mg of thiourea were dissolved in 30 mL Milli-Q water. The solution was transferred to a 50 mL autoclave, and was kept in an oven at 200 °C for 24 h to obtain the layered MoS_2_. Then, it was centrifuged with ethanol and Milli-Q water 3~4 times and dried in vacuum at 60 °C for 12 h to acquire black powder. In order to prepare MoS_2_/Au heterojunctions, typically, 500 μL MoS_2_ powder aqueous suspension (10 mg·mL^−1^) was diluted into 100 mL Milli-Q water under ultrasound at 25 °C, and then 8 mL AA (0.1 mol·L^−1^) and 10 mL HAuCl_4_·4H_2_O (0.1 wt. %) were rapidly added. After stirring (1200 rpm) the solution for 1 h at 25 °C, the as-prepared product was centrifuged 3~4 times to attain the MoS_2_/Au heterojunction catalyst. Surface coverages of Au nanoparticles on the MoS_2_ can be adjusted by the different adding ratios of MoS_2_ powder and HAuCl_4_·4H_2_O. The SEM and TEM images of the prepared MoS_2_ and MoS_2_/Au are shown in Figure A1.

### 2.3. Instrument Characterizations and Experimental Tests

A scanning electron microscope (SEM, Hitachi SU8010) and a transmission electron microscope (TEM, FEI Tecnai G220) were used to analyze the morphology of the samples. The X-ray powder diffractometer (XRD, Bruck D8 Advance) was used to determine the crystal form with a test range of 30–80° (2θ). The UV–Vis spectrometer (TU-1810, Beijing persee) was employed to measure the absorbance of the supernatant of the reaction solution.

The measuring process of electron spin resonance (ESR) spectroscopy were performed as follows. A volume of 5 mL of MB (10^−4^ mol·L^−1^) and 1 mL of H_2_O_2_ (30%) aqueous solution were mixed. Then, 100 μL of DMPO (0.1 mol·L^−1^) and 5 mg of MoS_2_/Au heterojunction catalyst were added to the mixed solution in turn. The mixed solution was immediately transferred to a capillary, filling the 2/3 of the total volume. The measurements were carried out on an ESR spectrometer (JES-X30, Japan) at room temperature under 1 MW microwave (0.915 GHZ).

**Figure A2:**
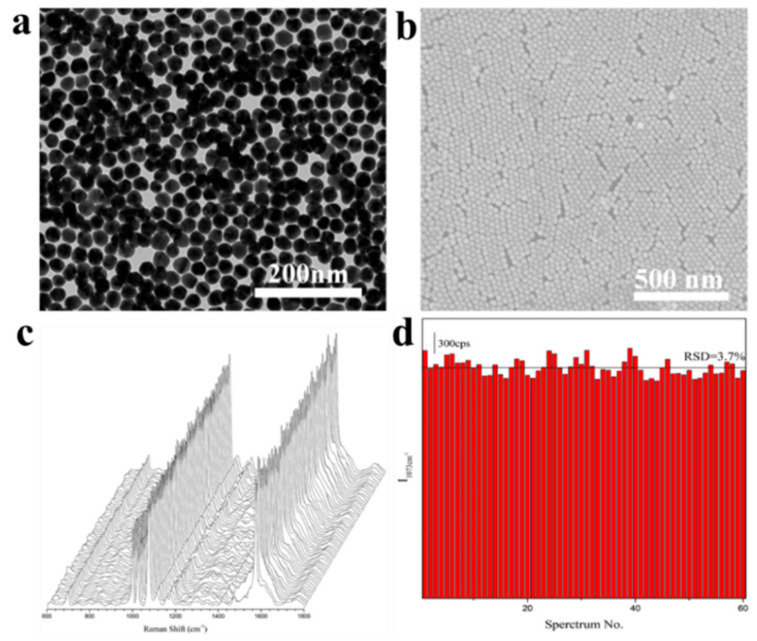
TEM and SEM images of 30 nm Au nanoparticle MLF (**a**,**b**); SERS of thiophenol on the Au nanoparticle MLF (**c**) and the intensities of SERS bands at 1073 cm^−1^ (**d**) on 60 points.

5 mg of MoS_2_/Au catalyst was added to 5 mL of MB (10^−4^ mol·L^−1^) and 1 mL of H_2_O_2_ (30%) aqueous solution. As the reaction proceeds, solutions of different reaction times are dropped onto the Au monolayer film (MLF, Figure A2) substrate, which is stuck on a Si wafer placed at the bottom of a solution container. After 10 min, the wafer was taken out and was dried for SERS detection. The SERS signals were recorded by a confocal Raman spectrometer with a 638 nm laser (Horiba Jobin Yvon, XploRA PLUS).

## 3. Results and Discussion

**Figure 1:**
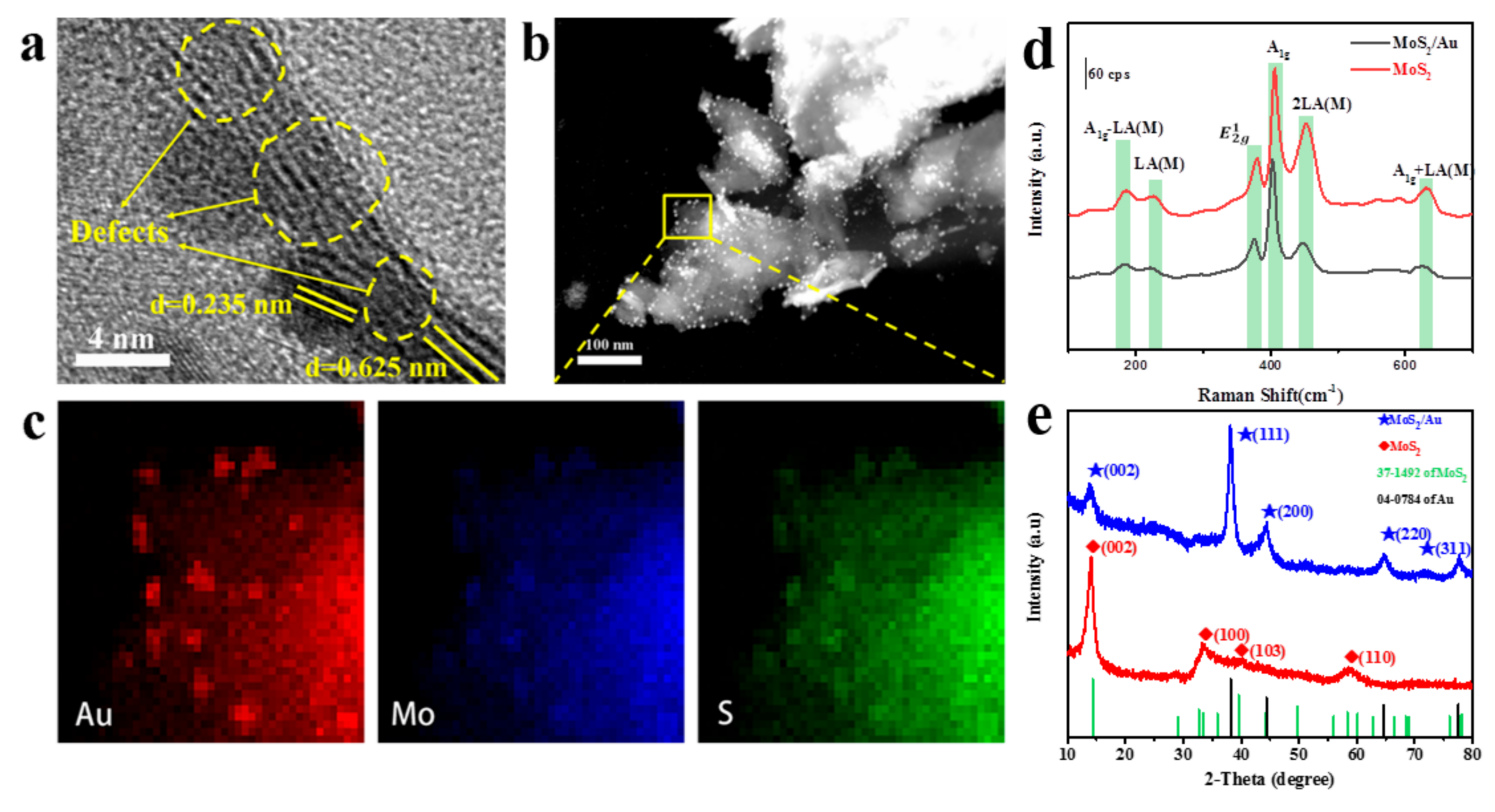
High resolution TEM image of MoS_2_/Au heterojunctions (**a**); HAADF-STEM image of MoS_2_/Au heterojunctions (**b**) and corresponding STEM-EDS element maps of Au, Mo, and S (**c**); Raman spectra (**d**); and XRD patterns (**e**) of MoS_2_ and MoS_2_/Au heterojunctions.

High resolution TEM was employed to observe the interface structure of MoS_2_/Au heterojunctions. It was reported that defects at the interface between Au and MoS_2_ can be formed due to their large difference in lattice constants and chemical composition, which was beneficial for improving the catalytic efficiency [25]. Figure 1a shows stripes with crystal plane spacings of 0.625 nm and 0.235 nm, which originated from the (002) plane of MoS_2_ and the (111) plane of Au, respectively [26]. It was observed that the lattice stripes of MoS_2_ were bent or interrupted to a certain extent, reflecting the defects of crystal lattice. Therefore, it was indicated that the introduction of Au nanoparticles produces more exposed high-energy and active Mo atoms, which laid the structure foundation for high catalytic efficiency from MoS_2_/Au [27]. We also employed high angle annular dark field scanning transmission electron microscopy (HAADF-STEM, Figure 1b) to observe MoS_2_/Au heterojunctions and selected local areas for energy dispersive X-ray element analysis (Figure 1c). It was found that in Figure 1c, the Au nanoparticles dispersed on the surface of MoS_2_. In addition, it also reveals uniform distribution of Mo and S atoms, in particular, shown as blue and green colors, respectively. By plotting the distribution of Au, Mo, and S elements in the materials, the successful preparation of MoS_2_/Au heterojunctions was confirmed.

As shown in Figure 1d, Raman spectrum was collected from the MoS_2_ powder. The characteristic bands can be detected at 183 cm^−1^, 223 cm^−1^, 380 cm^−1^, 404 cm^−1^, 453 cm^−1^, and 628 cm^−1^, which were assigned to vibration modes of A_1g_-LA (M), LA (M), $E_{2g}^{1}$, A_1g_, 2LA (M), and A_1g_ +LA (M), respectively [28]. It was worth pointing out that the bands at 380 cm^−1^ ($E_{2g}^{1}$) and 404 cm^−1^ (A_1g_) were generated from the in-plane and out-of-plane vibrations of Mo and S atoms. The wavenumber difference from two bands can be employed to evaluate the number of MoS_2_ layers [29]. Due to the Van der Waals forces and long-range Coulomb interlayer interactions [30], it has been reported that MoS_2_ stacks from a single layer to a multilayer with a wavenumber difference of 13–25 cm^−1^ [31,32]. In our case, the wavenumber difference is 24 cm^−1^, indicating that it was multilayered structure. The Raman spectrum collected from the MoS_2_/Au was almost the same comparing to that of MoS_2_. Therefore, we cannot detect Raman signals from Au nanoparticles themselves, although they can enhance the Raman signals from the probe molecules.

Figure 1e shows the XRD patterns of MoS_2_ and MoS_2_/Au heterojunctions. According to the JCPDS (Card No. 37-1492), the bands located at 14.4°, 32.7°, and 58.3° were ascribed to the (002), (100), and (110) crystal planes of MoS_2_, respectively. While the bands at 38.2°, 44.4°, 64.6°, and 77.5° originated from the (111), (200), (220), and (311) crystal planes of Au through the JCPDS (Card No. 04-0784). XRD pattern from MoS_2_/Au shows both the characteristic diffraction bands of MoS_2_ and Au, further indicating that Au nanoparticles were successfully coated on the surfaces of MoS_2_.

**Figure 2:**
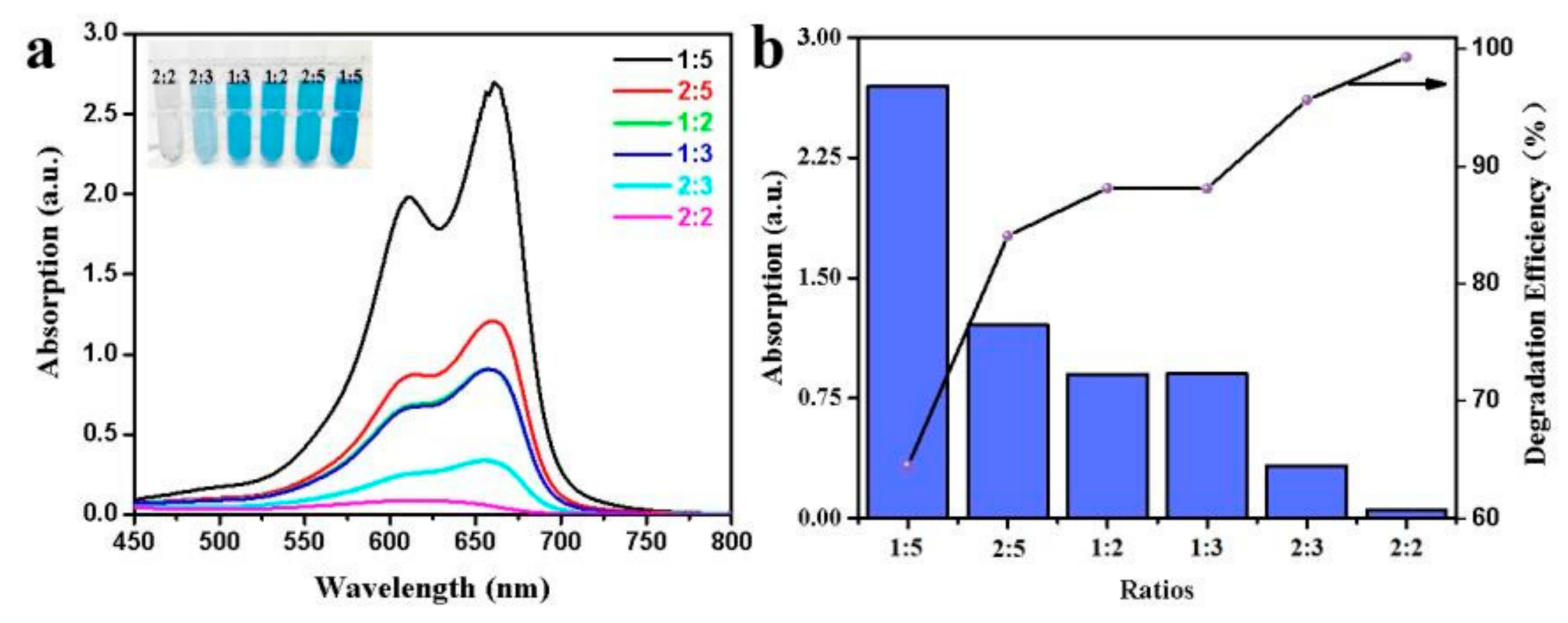
UV–vis absorption degradation curves of MB catalyzed by MoS_2_/Au at different addition ratios (MoS_2_ (mg): HAuCl_4_ (wt. % mL)) (**a**); Histogram of absorption intensity and corresponding catalytic efficiency curve (**b**).

**Figure A3:**
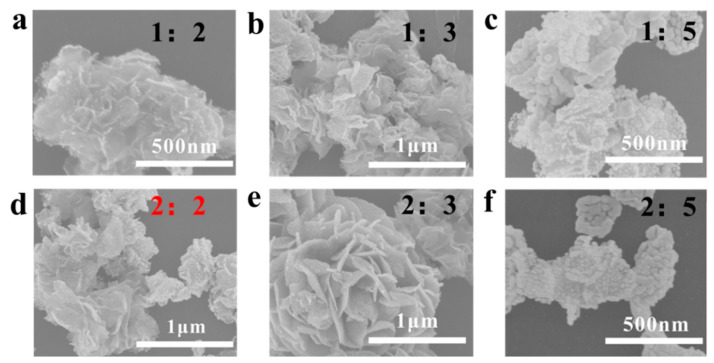
SEM images of MoS_2_/Au obtained from MoS_2_ and chloroauric acid at different addition ratios.

MoS_2_/Au heterojunctions with different Au coverages (six samples, Figure A3) were prepared to study the relationship between the ratios of Mo/Au and the catalytic effect. The Fenton-like reaction by using MB as a target degradation molecule was employed to estimate the catalytic performances of these MoS_2_/Au heterojunctions. The catalytic performances were judged by detecting the remaining MB concentration after keeping these reaction vessels for a long enough time (48 h). As shown in the inset image of Figure 2a, in fact, it can be intuitively observed by the naked eye that the optimal ratio is 2:2, implied by the colorless solution. However, it is difficult to evaluate the color change in a short time. Therefore, UV–visible spectrum measurements were performed. The maximum absorption wavelength was around 664 nm. The intensity of this absorption band was employed to evaluate the remaining concentration of MB. Formula (1) is used to calculate catalytic efficiency,

(1) $$\eta \left(\%\right)=\frac{C_{0}-C}{C_{0}}\times 100\%$$

where *η* stands for the catalytic efficiency, and *C*_0_ and *C* represent the initial and final concentrations of MB. It is shown in Figure 2a that the maximum absorption intensity is 2.7, while the minimum intensity is only 0.05, which quantitatively indicated the best performance of the reactant ratio (2:2). It is worth noting that the absorption band intensity at the beginning of the reaction is 7.6. Therefore, the catalytic efficiency calculated according to the absorption band intensity is 64.5%, 84.1%, 88.2%, 88.1%, 95.7%, and 99.3% (Figure 2b). It is well known that the edge-active Mo atoms and surface defects of MoS_2_ are beneficial to improve the catalytic performance [25]. Therefore, the coverage of Au nanoparticles on the surface is particularly critical. If the coverage of Au nanoparticles was too low, it was difficult to form hot spots for SERS detection as well as interfacial defects for efficient catalysis. Conversely, when there were too many Au nanoparticles on the surface, MoS_2_ would be completely coated, which only reflected the physicochemical properties of Au nanoparticles and lost the structure advantage of heterojunction [33]. Therefore, the catalyst (2:2) with the best catalytic performance was used in the subsequent study of SERS monitoring reaction.

**Figure 3:**
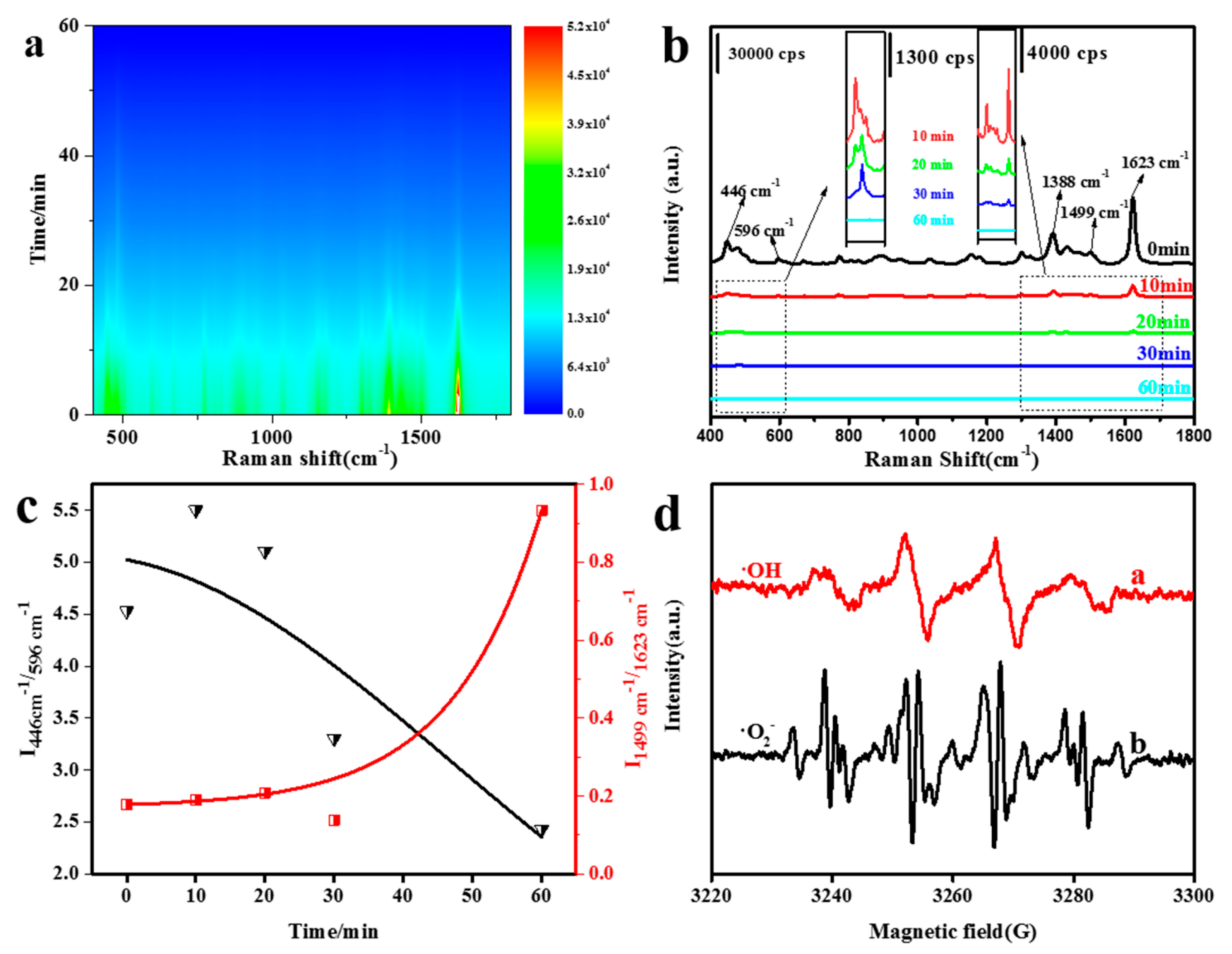
Time-dependent SERS mapping of catalytic degradation of MB by MoS_2_/Au heterojunctions (**a**); SERS spectra of catalytic degradation of MB by MoS_2_/Au heterojunctions (**b**); The relative intensities of SERS bands at 446 to 549 cm^−1^ and 1499 to 1623 cm^−1^ as a function of reaction time (**c**); ESR spectra of **·**OH and **·**O_2_^−^ in the catalytic degradation of MB by MoS_2_/Au using DMPO as the spin-trapper (**d**).

Comparing to UV–vis absorption spectroscopy [34], SERS owns ultra-high sensitivity and structural identification ability, exhibiting more advantages in monitoring the reaction process. Au nanoparticles on the MoS_2_ surface and the underlying Au monolayer film jointly built “hot spots”, which greatly enhanced the SERS signals. Figure 3a shows the time-dependent SERS mapping of residual MB signals. It was found that there were two strong peaks at 1388 cm^−1^ and 1623 cm^−1^, which were ascribed to the C-H and C-C ring stretching vibrations, respectively [35]. Since the band intensity at 1623 cm^−1^ was the largest, it was considered as the benchmark for evaluating the catalytic efficiency.

Figure 3b shows the time-dependent SERS spectra on the Fenton-like reaction. Before the start of the reaction (0 min), a strong SERS signal of MB can be detected. Then, H_2_O_2_ and MoS_2_/Au were added to 5 mL of 10^−4^ mol·L^−1^ MB solution. It was found that the MB signals dropped 83% within 10 min. After 30 min, the band intensity dropped 97%, and the catalytic reaction was almost completed at 60 min. In addition to the intensity change, SERS can also supply the information from molecular structure. The bands located at 446 cm^−1^, 596 cm^−1^, and 1499 cm^−1^ corresponded to the C-N-C skeletal vibration, C-S-C symmetrical stretching vibration, and –NH_2_ deformation vibration, respectively [36]. The inset images were the partial enlarged view of two spectra ranged from 400 cm^−1^ to 600 cm^−1^ and 1300 cm^−1^ to 1700 cm^−1^. It was reported that the information of molecular structure changes in the degradation process can be obtained through the change of relative band intensity. For example, Zhang’s group designed “hedgehog-like” Ag nanocones on polystyrene microsphere arrays and studied the plasmon-induced photocatalytic degradation process of MB [37]. The change of spectral intensity in their study revealed the degradation mechanism of MB molecules. In our case, the value of I_446cm_^−1^/I_596cm_^−1^ decreased from 4.5 to 2.5 within 60 min in Figure 3c, indicating that the C-N-C bond was easier to break than the C-S-C bond. The value of I_1499_cm^−1^/I_1623cm_^−1^ remained almost stable in the first 30 min of the reaction, indicating that –NH_2_ group and the fused ring were destroyed at the comparable speed. At 60 min, the ratio increased significantly, which may be due to the coordination and retention of the –NH_2_ group with the metal surface, while the fused ring was seriously damaged, resulting in the change of the relative intensity of the two bands.

To further study the reaction mechanism, ESR spectroscopy was used to monitor the reaction intermediates. DMPO is often used as a scavenger for hydroxyl radicals. When **·**OH is present, DMPO combines them to form DMPO-**·**OH adducts. As shown in Figure 3d, four characteristic bands with spectral intensity ratios of 1:2:2:1 were observed, indicating that **·**OH existed in the system. It was in agreement with the previously reported DMPO-**·**OH addition [38]. Meanwhile, the DMSO solution of DMPO can be used as a capture agent for superoxide radical anion (**·**O_2_^−^). When **·**O_2_^−^ is present, DMPO combines with them to form DMPO-**·**O_2_^−^ adduct. The characteristics of ESR spectrum was consistent with those reported in other literatures [39]. **·**O_2_^−^ was more reactive than dissolved oxygen in aqueous solution because its π* orbital contained unpaired electrons [40]. The unpaired electrons of negatively charged **·**O_2_^−^ can be used as highly active substances to initiate free radical reactions or induce single electron reduction/oxidation reactions.

**Figure 4:**
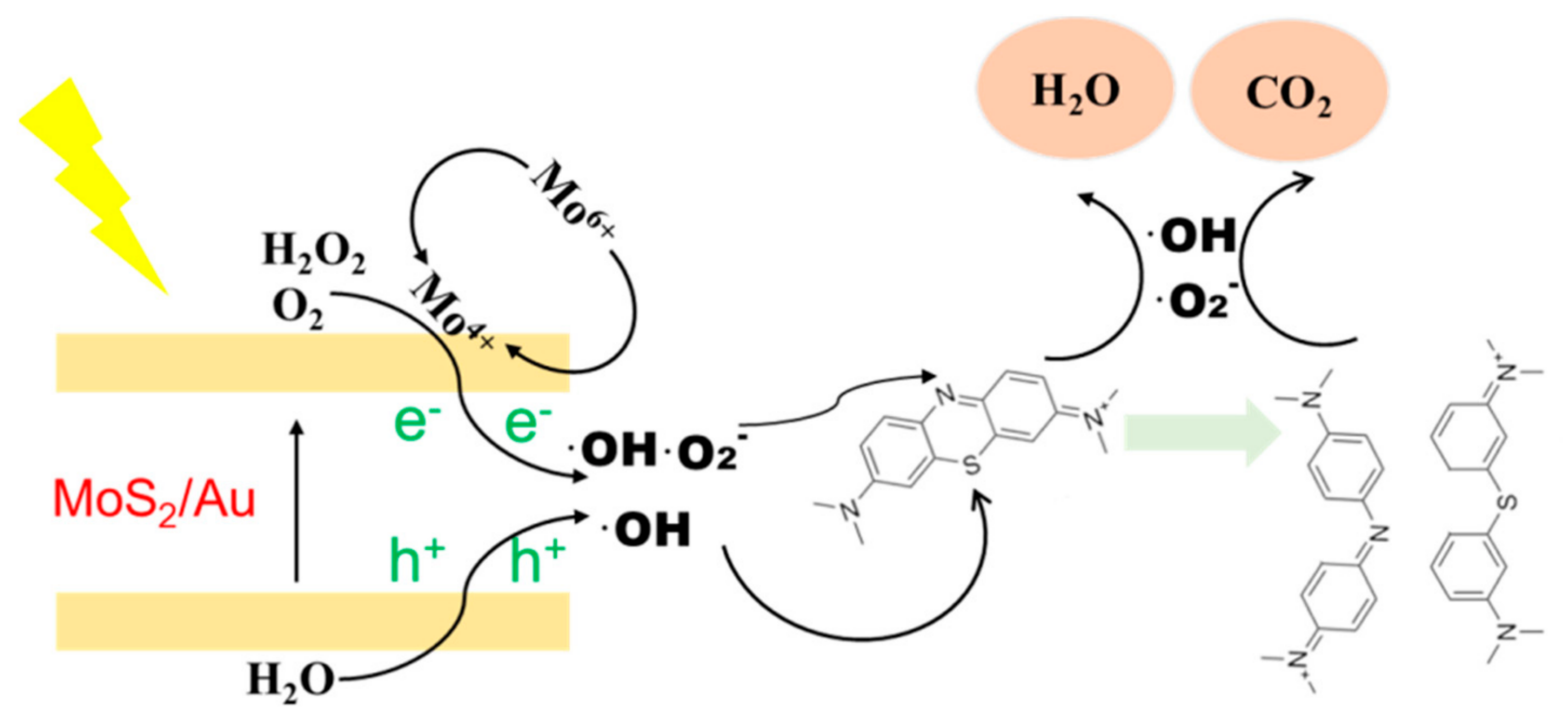
Schematic diagram of the possible Fenton-like catalytic mechanism of MoS_2_/Au.

Therefore, the **·**OH and **·**O_2_^−^ produced in the Fenton-like reaction catalyzed by MoS_2_/Au heterojunctions were considered to be the main contributors to the degradation of MB. A possible reaction mechanism was proposed for the Fenton-like system with MoS_2_/Au as catalyst, as shown in Figure 4 and the following chemical Equations (2)–(8).

MoS_2_/Au + *hv* → e^−^ + h^+^ (2)

H_2_O + h^+^ → **·**OH + H^+^ (3)

H_2_O_2_+ e^−^ → **·**OH + OH^−^ (4)

O_2_ + e^−^ → **·**O_2_^−^ (5)

Mo^4+^+H_2_O_2_ → Mo^6+^+OH^−^+**·**OH (6)

Mo^6+^ + e^−^ → Mo^4+^ (7)

**·**OH, **·**O_2_^−^ + Dye → H_2_O + CO_2_ (8)

Under natural light, holes (h^+^) and electrons (e^−^) were produced in the reaction solution due to the existence of MoS_2_/Au heterojunctions. Holes and electrons can react with water, hydrogen peroxide, and dissolved oxygen to form **·**OH or **·**O_2_^−^ [41]. The Mo^4+^ and Mo^6+^ formed a cycle and produced **·**OH at the same time [42]. Both **·**OH and **·**O_2_^−^ have oxidation and can eventually mineralize MB into CO_2_ and water.

**Figure 5:**
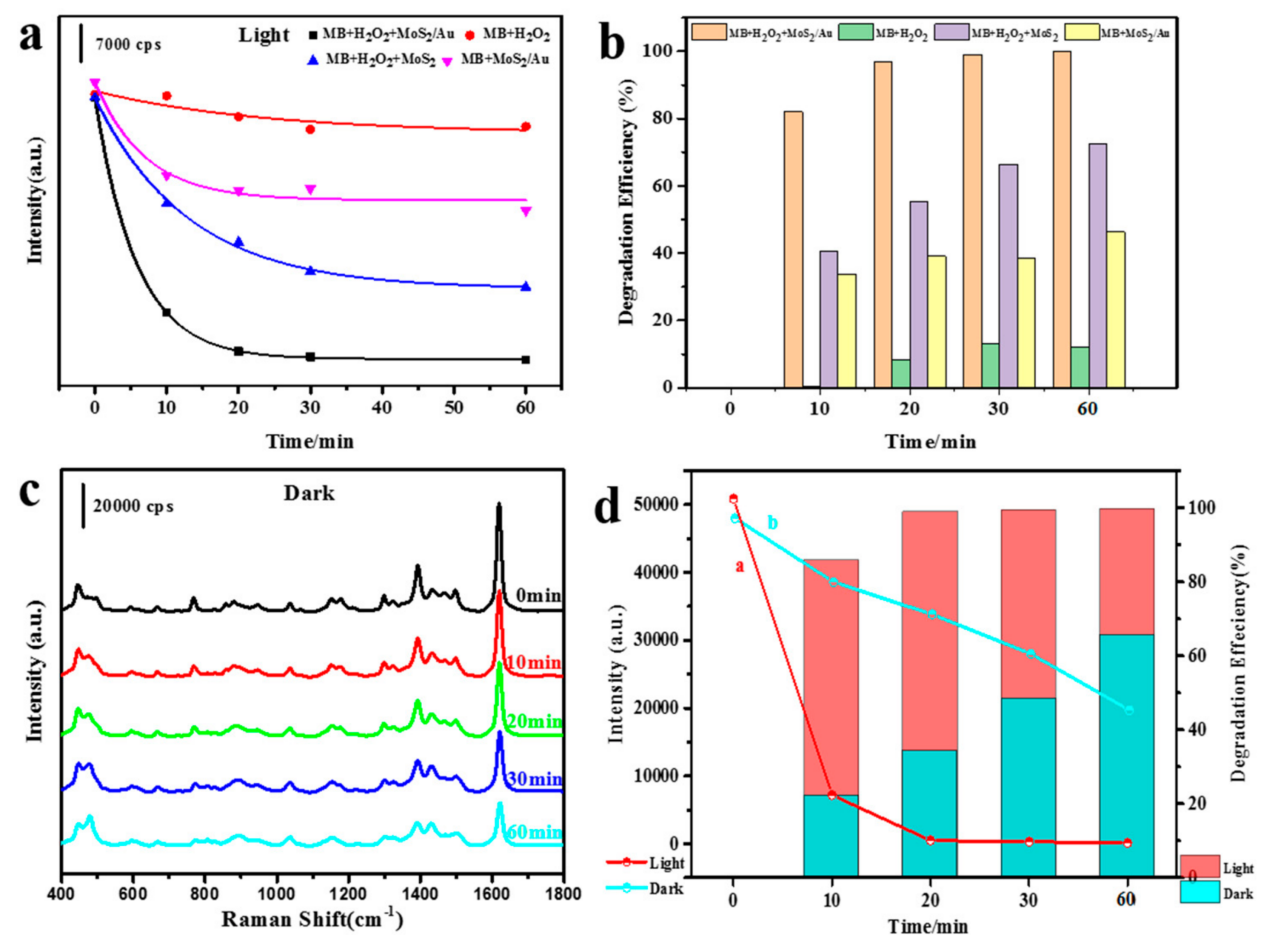
SERS intensity from the band at 1623 cm^−1^ as a function of reaction time catalyzed by MoS_2_/Au or MoS_2_ in different conditions under natural light (**a**) and corresponding catalytic efficiencies (**b**); Time-dependent SERS of degradation of MB by MoS_2_/Au (in dark) (**c**); Time-dependent SERS intensities and degradation efficiencies in dark and natural light (**d**).

Figure 5a,b present control experiments on the degradation of MB based on the band intensity at 1623 cm^−1^ under various conditions. It was observed that the SERS intensity was reduced about 10% within 60 min when there were only MB and H_2_O_2_. It was revealed that no Fenton-like reaction occurred when MoS_2_/Au was absent. When only MB and MoS_2_/Au were present, the SERS band intensity was reduced about 45%. This can be mainly attributed to the fact that the layered MoS_2_ provided a large surface area for MB adsorption. This is due to the physical interaction, not the actual degradation of MB. However, when MB, H_2_O_2_ and MoS_2_ were mixed, a Fenton-like reaction could occur. The degradation rate reached 70% in 60 min. To further improve the catalytic efficiency, it was necessary to replace MoS_2_ with MoS_2_/Au. It was revealed that 83% of degradation efficiency was achieved at 10 min and approached 100% at 60 min. This demonstrated that the catalytic performance of MoS_2_/Au was much better than that of MoS_2_. We considered that the surface defects produced by the heterojunction structure facilitated the catalytic reaction process.

Generally, the light-assisted Fenton reaction has a faster rate and a higher degree of mineralization than the thermal (dark) reaction [43]. In order to study the effect of light on the Fenton-like reaction, the control experiments under dark condition were performed. As shown in Figure 5c, the degradation efficiency was only 22% in dark at 10 min comparing to that of 83% under the natural light, indicating that natural light significantly improved the catalytic efficiency. It is worth noting that the natural light mentioned here does not use special light sources, such as xenon lamps, but only the light obtained in the natural environment of the laboratory. This result demonstrated that our MoS_2_/Au catalyst had tremendous advantages compared with those catalysts using visible light [44] or UV lamp irradiation [45], in which existed huge differences in actual application environments. Figure 5d further compares the band intensity and degradation efficiency in dark and natural light conditions. It was found that the natural light obviously accelerated the reaction process. Specially, we can observe that the band intensity decreased from about 50,000 to 40,000 in the first 10 min in the dark condition. However, it rapidly decreased below 10,000 in the presence of natural light. The histogram reflected the differences in degradation efficiencies. At each time node within 60 min, the catalytic efficiencies in the presence of natural light were always higher than that in the dark environment.

**Figure 6:**
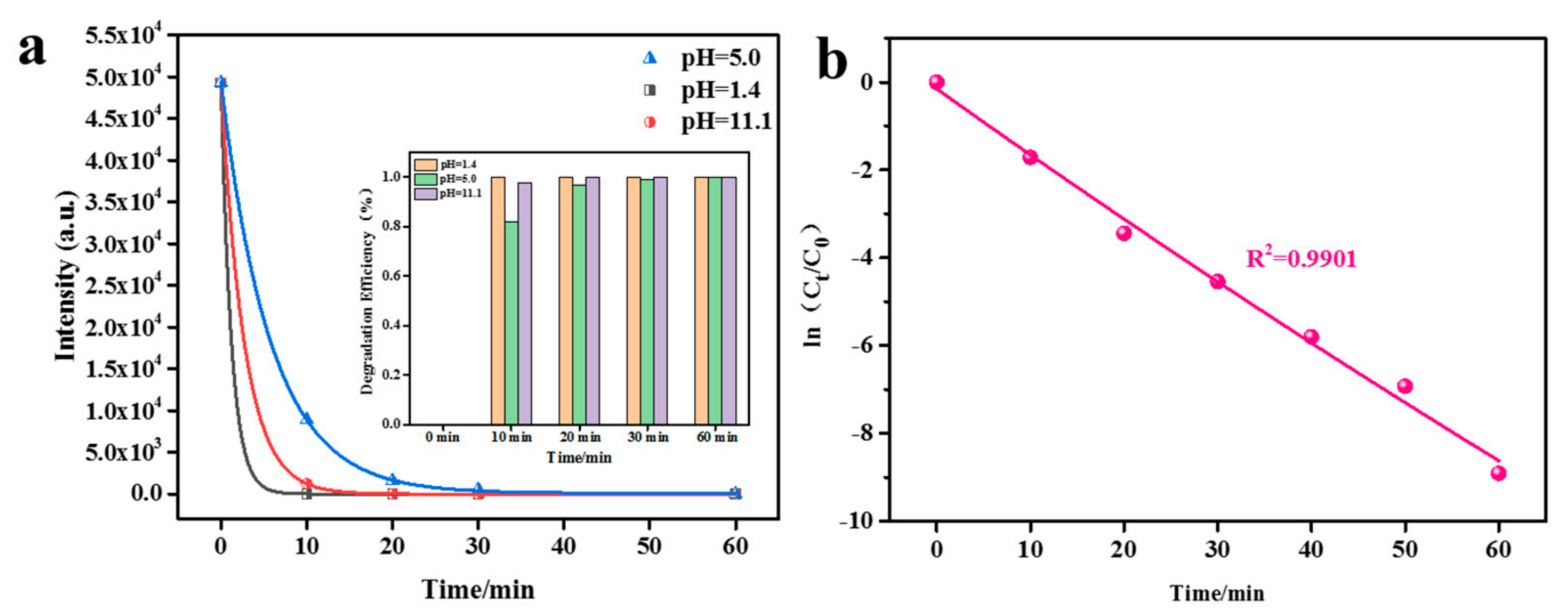
SERS intensity and efficiency (inset) diagrams of MB at band of 1623 cm^−1^ catalyzed by MoS_2_/Au as a function of reaction time at different initial pH values (**a**); Kinetic fitting curve of SERS intensity of MB at band of 1623 cm^−1^ as a function of reaction time (**b**).

In order to further exploit the advantages of this catalyst, we tried to study the application scope of the catalyst. For a classic Fenton process, Fenton’s reagent has a strong oxidative ability and can rapidly degrade organic matter [46]. However, the pH range used was relatively narrow, usually 2–4. If the solution pH value was not within this range, it would either hinder the circulation of iron or inhibit the formation of active free radicals, and might also cause ferric hydroxide precipitation. Therefore, the classical Fenton reaction system has great limitations in the applicability of catalytic objects. For our cases, at different pH values, hydrochloric acid and sodium hydroxide were employed to adjust the pH values to 1.4 and 11.1, respectively. As shown in Figure 6a, when the pH was 1.4 and 11.1, the band intensity at 1623 cm^−1^ sharply decreased, and its intensity changed from 49,287 cps to 16 cps and 1230 cps at 10 min, respectively. This shows that the catalytic efficiency under acidic conditions is better than that under alkaline conditions. Interestingly, we can observe that the blue line is above the red and black line, which shows that compared with natural conditions (without any acid-base regulator, pH = 5), both strongly acidic and alkaline environments are conducive to improving the catalytic efficiency. As shown from inset histogram in Figure 6a, the degradation efficiencies of MB in all three pH values reached more than 95% within 20 min, although the experiment lasted for 60 min. This not only shows that our catalyst has a wide range of pH application range, but also has achieved good catalytic results in such a wide range.

In general, the SERS intensity is proportional to the molecular concentration with the same SERS substrate. The intensity of the characteristic band at 1623 cm^−1^ were used to represent the concentration change of MB solution. The first-order kinetic curve is shown in Equation (9):

(9) $$\ln\frac{C_{t}}{C_{0}}=-kt$$

Here, *C_t_* was the concentration of MB at the reaction time *t*. *k* was the reaction rate constant, and *t* was the reaction time. As can be seen from Figure 6b, ln (*C_t_*/*C*_0_) has a linear relationship with the reaction time *t*, and the rate constant *k* was 0.146 min^−1^, following the first-order kinetic process. The regression coefficient value (R^2^) of the fitted curve is 0.9901. The result indicates that the Fenton-like catalytic degradation of MB was a first-order kinetic reaction, which is similar to that reported in the literature [47,48]. Therefore, through SERS technology, we can not only monitor the process of catalytic reaction, but also analyze it from the perspective of kinetics and molecular structure, so as to lay a foundation for revealing the mechanism of complex reactions.

## 4. Conclusions

Advanced oxidation process has played an important role in environmental pollution control. In this paper, the layered two-dimensional material MoS_2_ is combined with Au nanoparticles with SERS effect to construct the MoS_2_/Au heterojunctions, which have both catalytic and SERS detection capabilities. The introduction of Au nanoparticles also further increases the surface defects of MoS_2_, which is more conducive to improving the catalytic efficiency. Unlike many studies using MoS_2_ as a cocatalyst, in this study, MoS_2_ was used as the main catalyst, so there was no iron element in the system, and there was no disturbing iron sludge, which greatly broke through the limitations of the classical Fenton reaction.

As a highly sensitive technology, SERS monitored the degradation of MB in the whole process. SERS technology can not only effectively identify the degradation process of MB in terms of intensity and calculate the degradation efficiency, but also carry out preliminary structural change analysis. This is not available for most studies using UV–Vis spectroscopy as a detection method. Structural analysis shows that C-N-C bond was easier to break than the C-S-C bond. The NH_2_ group and the fused ring were destroyed at the comparable speed at the first 30 min. In terms of catalytic efficiency, the MB molecule was degraded by 83% in the first 10 min, and was completely degraded by 60 min. The contrast experiment shows that the catalytic efficiency can be significantly improved in the natural light of the laboratory when compared to complete darkness. The reaction can be carried out efficiently without special light sources, including visible light and ultraviolet light sources with high power. This laid a good foundation for the practical application of the catalyst in the open air. At the same time, SERS experiments also showed that the catalyst can play a very good role at pH 1.4, 5.0, and 11.1, thus breaking the limitation that many iron-based Fenton reactions can only be applied in the narrow range of pH 2–4. This study also showed that the catalytic reaction of MB was a first-order reaction. ESR experiments confirmed that both ·OH and ·O_2_^−^ promoted the reaction efficiency. Using SERS to study the reaction mechanism at the molecular level, the reusability and stability of the catalyst need to be further studied. Due to the increasingly miniaturization and cheapness of Raman spectroscopy instruments, the combination of SERS and catalytic reaction monitoring is not only conducive to the development of the SERS technology itself, but also can promote Raman spectroscopy technology to gradually enter all walks of life.

## Data Availability

All the data used in this study are already included in the manuscript.

## Appendix A

### Appendix A.1. Characterizations of MoS_2_ and MoS_2_/Au

As shown in Figure A1a,b, the hydrothermal synthesized MoS_2_ was a 2D irregular sheet structure, with a size between 300 nm and 500 nm. Part of sheets were assembled in the crisscross way. The adjacent sheets cross each other to form flower-shaped particles (FSPs, inset in Figure A1b) with the size of micron level, i.e., MoS_2_ micro–nano hierarchical structure. The Au nanoparticles with diameter of about 10 nm were formed on the sheets and FSP surface by one-step reduction of HAuCl_4_·4H_2_O, obtaining MoS_2_/Au heterojunctions. As shown in Figure A1c,d, Au nanoparticles grew on the MoS_2_ surface with a high density.

### Appendix A.2. Preparation of Au Monolayer Film (MLF)

15 nm Au seeds were prepared by the classical Frens one-step method. A volume of 100 mL of 0.01% chloroauric acid solution was heated to boiling under vigorous stirring. After that, 2 mL of 1% (*w*/*v*) trisodium citrate was quickly added, and the color changed to red and then continued to heat for 15 min. After the heating stopped, the Au seed solution continued to stir to room temperature.

1 mL of 1% (*w*/*v*) Na_3_C_6_H_5_O_7_, 20 mL of 25 mmol·L^−1^ NH_2_OH·HCl, and 1 mL of 1% (*w*/*v*) PVP (Mw ≈ 10 000) were added to the above 25 mL of 15 nm Au seed solution with stirring. Then, 20 mL of 0.1% chloroauric acid solution was dropwise added to the above solution by injection pump at a rate of 60 mL/h. After completing the injection, stirring continued for 1 h to obtain Au nanoparticles with an average diameter of 30 nm.

3 mL of the above 30 nm Au solution was added to the volatilization device and evaporated at 40 °C for 16 h. The Au MLF formed at the gas–liquid interface was transferred to a silicon wafer or copper mesh. The morphology and SERS performance for uniformity of Au MLF are shown in Figure A2.

### Appendix A.3. Control Au Coverages on the MoS_2_ Surface

MoS_2_/Au heterojunctions with different Au coverages were prepared by adjusting the amount of MoS_2_ (mg) and HAuCl_4_·4H_2_O (wt. % mL). Other conditions were controlled as follows: 20 mL ultrapure water and 2 mL 0.1 mol·L^−1^ AA. As shown in Figure A3, they were labeled as heterojunctions a to f.
